# The Risk of Cancer in Patients with Generalized Anxiety Disorder: A Nationwide Population-Based Study

**DOI:** 10.1371/journal.pone.0057399

**Published:** 2013-02-27

**Authors:** Cheng-Che Shen, Yu-Wen Hu, Li-Yu Hu, Man-Hsin Hung, Tung-Ping Su, Min-Wei Huang, Chia-Fen Tsai, Shuo-Ming Ou, Sang-Hue Yen, Cheng-Hwai Tzeng, Tzeon-Jye Chiou, Tzeng-Ji Chen, Chia-Jen Liu

**Affiliations:** 1 Department of Psychiatry, Chiayi Branch, Taichung Veterans General Hospital, Chiayi, Taiwan; 2 School of Medicine, National Yang-Ming University, Taipei, Taiwan; 3 Cancer Center, Taipei Veterans General Hospital, Taipei, Taiwan; 4 Institute of Public Health, National Yang-Ming University, Taipei, Taiwan; 5 Department of Psychiatry, Yuli Veterans Hospital, Hualian, Taiwan; 6 Division of Hematology and Oncology, Department of Medicine, Taipei Veterans General Hospital, Taipei, Taiwan; 7 Department of Psychiatry, Taipei Veterans General Hospital, Taipei, Taiwan; 8 Division of Nephrology, Department of Medicine, Taipei Veterans General Hospital, Taipei, Taiwan; 9 Department of Biomedical Imaging and Radiological Sciences, National Yang-Ming University, Taipei, Taiwan; 10 Department of Family Medicine, Taipei Veterans General Hospital, Taipei, Taiwan; 11 Department of Internal Medicine, National Yang-Ming University Hospital, Yilan, Taiwan; University of Sydney, Australia

## Abstract

**Objective:**

To evaluate the risk of cancer among patients with generalized anxiety disorder (GAD) in a nationwide population-based dataset.

**Methods:**

We recruited newly-diagnosed GAD patients aged 20 years or older without antecedent cancer from the Taiwan National Health Insurance Research database between 2000–2010. Standardized incidence ratios (SIRs) of cancers were calculated in GAD patients, and the subgroup of GAD patients diagnosed by psychiatric specialists.

**Results:**

A total of 559 cancers developed among 19,793 GAD patients with a follow-up of 89,485 person-years (median follow-up of 4.34 years), leading to a significantly increased SIR of 1.14 [95% confidence interval (CI) 1.05–1.24]. Male GAD patients had a significantly increased SIR overall (1.30, 95% CI 1.15–1.46) and for lung and prostate cancer (1.77, 95% CI 1.33–2.30 and 2.17, 95% CI 1.56–2.93, respectively). Patients over 80 years of age also had a significantly increased SIR (1.56, 95% CI 1.25–1.92), especially in males. However, psychiatrist-diagnosed GAD patients did not show increased cancer risk relative to the general population, perhaps due to having fewer physical comorbidities than non-psychiatrist-diagnosed GAD patients.

**Conclusion:**

This study found that overall cancer risk is elevated among patients with GAD. The risk of lung and prostate cancer also increased in male patients with GAD. This increased cancer risk may be due to physical comorbidities and surveillance bias. Further prospective study is necessary to confirm these findings.

## Introduction

Generalized anxiety disorder (GAD) is one of the most common anxiety disorders among the general population. It is characterized by persistent and excess worrying, sleep disturbance, muscle tension and being easily fatigued. [1] According to two major studies in the United States, the Epidemiological Catchment Area study and the National Comorbidity Survey study, the estimated lifetime prevalence rate for GAD is 4.1–6.6%. [2], [3] In a large cohort study in Germany, the 12-month prevalence rate of GAD in the community was 3.0%. [4].

Despite the availability of several treatment options, GAD remains a major public health concern. It is consistently associated with considerable disability and overutilization of medical services. [5] GAD has a detrimental impact on patients’ work, education, social relationships and quality of life. [6], [7] It is also associated with negative health outcomes, such as increased risk for cardiovascular events and premature death in patients with chronic obstructive pulmonary disease (COPD). [8]–[11] Therefore, GAD incurs considerable costs due to healthcare expenses and loss of productivity. [12].

Psychological distress, such as ongoing depression and anxiety-related symptomatology, has been associated with poorer survival rate of cancer patients. [13], [14] However, whether psychological factors increase the incidence of cancer is unclear. Numerous papers in the literature investigated associations between cancer incidence and psychosocial factors, such as anxiety, personality and depression. However, the results are controversial, [14]–[24] and few of these studies focused on anxiety or anxiety disorders. [20]–[22] A study in Taiwan found that patients with anxiety disorders had an increased risk of developing prostate cancer, a marginally decreased risk of cervical cancer, but no increase in overall cancer risk. [20] In addition, a study in the UK showed that patients with anxiety disorders had higher risks for lung and brain cancer, but no increase in overall cancer risk. [21].

The aim of the current study was to determine whether GAD is associated with an increased risk of cancer. This study was a population-based retrospective cohort study using a database derived from the National Health Insurance (NHI) system in Taiwan.

## Materials and Methods

### Data Sources

The NHI program, which began in 1995, is a mandatory universal health insurance program, offering comprehensive medical care coverage to all Taiwanese residents with a coverage rate of up to 98%. [25], [26] It includes coverage for outpatient, inpatient, emergency, dental, and traditional Chinese medicine services, as well as prescription drugs. This study used the NHI research database, which is managed and made available to the public by the National Health Research Institute (NHRI) of Taiwan. The NHI database of catastrophic illness provides comprehensive enrollment information for all patients with severe diseases, such as cancer, who have received copayment exemption under the NHI program. Confidentiality is maintained according to the data regulations of the Bureau of National Health Insurance and the NHRI. This study was exempt from full review by the institutional review board, as the NHI dataset consists of de-identified secondary data for research purposes.

### Study Population

We conducted a retrospective cohort study from January 1, 1996 to December 31, 2010. Newly-diagnosed patients with GAD were selected from January 1, 2000 to December 31, 2009. This study recruited patients aged 20 years or older at the time of GAD diagnosis who had no prior malignancies. GAD was defined according to the International Classification of Diseases, Ninth Revision, Clinical Modification (ICD-9-CM) code 300.02.

### Statistical Analyses

The main dependent variable was occurrence of cancer, as reported in the Registry for Catastrophic Illness. For a diagnosis of cancer to be reported in the Registry, histological confirmation is required. GAD patients were followed until development of cancer, death, dropout from the NHI program, or the end of the year 2010.

The risk of cancer among the GAD cohort was determined using a standardized incidence ratio (SIR), which is defined as the observed number of cancer occurrences divided by the expected number. The expected number of cancers was calculated by multiplying the national incidence rate of cancers, stratified by gender, calendar year, and age in 5-year intervals, by the corresponding stratum-specific person-time accrued in the cohort. The incidence rates of cancers among the general population were obtained from the Taiwan Cancer Registry. The 95% confidence intervals (CIs) for the SIRs were estimated under the assumption that the number of cancers followed a Poisson probability distribution. We determined the SIRs for subgroups for gender and age. To investigate potential surveillance bias and the possibility of GAD as a paraneoplastic phenomenon, subgroups were stratified by the duration since GAD diagnosis. SIRs were also estimated for each cancer type. Finally, SIRs for the subgroup of GAD patients diagnosed by psychiatric specialists were determined. Age at diagnosis and prevalence of physical comorbidities before GAD diagnosis were also analyzed. Categorical variables were compared using chi-square tests, and continuous variables were compared using t-tests.

Data extraction and computations were performed using the Perl programming language (version 5.12.2). Microsoft SQL Server 2005 (Microsoft Corp., Redmond, WA, USA) was used for data linkage, processing, and sampling. All statistical analyses were performed using SPSS statistical software version 17.0 for Windows (SPSS, Inc., Chicago, Illinois). Statistical significance was defined as a *p* value of less than 0.05.

## Results

### Patient Demographics

Overall, the cohort was observed for 89,485 person-years from 2000–2010. A total of 19,793 patients with GAD (64.4% female) were identified. The mean age at diagnosis was 49.6 years (standard deviation, 15.7 years), and the median follow-up period was 4.34 years (interquartile range, 2.1–6.8 years). Patient demographics are shown in Table 1.

**Table 1:** Characteristics of patients with generalized anxiety disorder.

	Total	Male	Female
No. of patients	19793	7041	12752
Person-years at risk	89484.9	31437.3	58047.6
Median follow-up, years (interquartile range)	4.3(2.1–6.8)	4.3 (2.1–6.7)	4.4 (2.2–6.8)
Mean age at diagnosis(±SD), years	49.6 (±15.7)	49.9 (±16.3)	49.5 (±15.4)
Distribution of patients according to age			
20–39	5832	2159	3673
40–59	8748	2887	5861
60–79	4654	1754	2900
≥80	559	241	318

SD standard deviation;

### All Cancers

A total of 559 cancers occurred within the observation period. Compared with the general population, patients with GAD had an increased overall cancer risk with an SIR of 1.14 (95% CI 1.05–1.24). A subgroup analysis for gender showed that males had an increased SIR (1.30, 95% CI 1.15–1.46, *p*<0.001), but females did not (1.02, 95% CI 0.91–1.15). A subgroup analysis for age showed that patients aged 80 years and older had an increased SIR (1.56, 95% CI 1.25–1.92, *p*<0.001), but those under 80 did not. Males over 80 years of age showed the highest SIR (1.82, 95% CI 1.35–2.39, *p*<0.001). Within the first year of GAD diagnosis, 111 cancers occurred with an SIR of 1.22 (95% CI 1.01–1.47, p = 0.044). If the first year was excluded, the SIR remained significantly elevated at 1.12 (95% CI 1.02–1.23, p = 0.015). The results of the subgroup analysis are summarized in Table 2.

**Table 2:** Standardized incidence ratios (SIRs) according to age, gender and duration of generalized anxiety disorder.

	Total			Male			Female		
Characteristics	Observed	Expected	SIR (95% CI)	Observed	Expected	SIR (95% CI)	Observed	Expected	SIR (95% CI)
All cancers	559	489.07	1.14(1.05–1.24)	274	210.65	1.30(1.15–1.46)	285	278.42	1.02(0.91–1.15)
Age, years									
20–39	24	19.18	1.25(0.80–1.86)	10	5.91	1.69(0.81–3.11)	14	13.27	1.05(0.58–1.77)
40–59	167	163.89	1.02(0.87–1.19)	64	54.70	1.17(0.90–1.49)	103	109.18	0.94(0.77–1.14)
60–79	282	250.77	1.12(1.00–1.26)	149	121.96	1.22(1.03–1.43)	133	128.82	1.03(0.86–1.22)
≥80	86	55.23	1.56(1.25–1.92)	51	28.09	1.82(1.35–2.39)	35	27.14	1.29(0.90–1.79)
Duration									
0–1	111	90.83	1.22(1.01–1.47)	53	39.07	1.36(1.02–1.77)	58	51.76	1.12(0.85–1.45)
≥1	448	398.25	1.12(1.02–1.23)	221	171.58	1.29(1.12–1.47)	227	226.66	1.00(0.88–1.14)
1–5	309	275.93	1.12(1.00–1.25)	154	118.78	1.30(1.10–1.52)	155	157.16	0.99(0.84–1.15)
≥5	139	122.31	1.14(0.96–1.34)	67	52.81	1.27(0.98–1.61)	72	69.51	1.04(0.81–1.30)

SIR Standardized incidence ratio; CI confidence interval.

### Specific Cancer Types

The cancer types most commonly observed in the GAD cohort were lung and mediastinum (n = 90), followed by liver and biliary tract (n = 82), then colon and rectum (n = 79). An increased SIR was observed for lung and mediastinum (1.53, 95% CI 1.23–1.88, *p*<0.001) and prostate cancers (2.17, 95% CI 1.56–2.93, *p*<0.001) in GAD patients. However, the increased SIR of lung and mediastinum was observed only in male patients (1.77, 95% CI 1.33–2.30, *p*<0.001), but not in females (1.27, 95% CI 0.88–1.76). SIRs for specific types of cancers are presented in Table 3. If the person-time of the first year was excluded, SIRs of lung cancer (1.49, 95% CI 1.16–1.89, *p* = 0.002) and prostate cancer (2.24, 95% CI 1.57–3.10, *p*<0.001) remained significantly elevated. Excluding patients with prostate or lung cancer did not increase the risks for other types of cancers in the total GAD cohort (1.04, 95% CI 0.94–1.14) or in the subgroup of males with GAD (1.10, 95% CI 0.95–1.28).

**Table 3:** Standardized incidence ratios (SIRs) for specific cancer types among patients with generalized anxiety disorder.

	Total			Male			Female		
Site of cancers	Observed	Expected	SIR (95% CI)	Observed	Expected	SIR (95% CI)	Observed	Expected	SIR (95% CI)
All cancers	559	489.07	1.14(1.05–1.24)	274	210.65	1.30(1.15–1.46)	285	278.42	1.02(0.91–1.15)
Head and neck	30	37.01	0.81(0.55–1.16)	24	28.79	0.83(0.53–1.24)	6	8.21	0.73(0.27–1.59)
Digestive	204	181.31	1.13(0.98–1.29)	108	92.81	1.16(0.95–1.40)	96	88.50	1.08(0.88–1.32)
Esophagus	5	8.36	0.60(0.19–1.40)	5	7.26	0.69(0.22–1.61)	0	1.10	0.00(0.00–3.37)
Stomach	30	24.08	1.25(0.84–1.78)	17	12.50	1.36(0.79–2.18)	13	11.59	1.12(0.60–1.92)
Colon and rectum	79	71.31	1.11(0.88–1.38)	38	31.19	1.22(0.86–1.67)	41	40.12	1.02(0.73–1.39)
Anus	0	0.52	0.00(0.00–7.15)	0	0.17	0.00(0.00–21.70)	0	0.35	0.00(0.00–10.66)
Liver andbiliary tract	82	68.25	1.20(0.96–1.49)	45	37.82	1.19(0.87–1.59)	37	30.43	1.22(0.86–1.68)
Pancreas	8	8.79	0.91(0.39–1.79)	3	3.87	0.78(0.16–2.27)	5	4.92	1.02(0.33–2.37)
Lung andmediastinum	90	58.75	1.53(1.23–1.88)	55	31.09	1.77(1.33–2.30)	35	27.66	1.27(0.88–1.76)
Bone and Softtissue	7	3.79	1.85(0.74–3.80)	5	1.73	2.90(0.94–6.76)	2	2.07	0.97(0.12–3.50)
Skin	8	9.57	0.84(0.36–1.65)	3	3.88	0.77(0.16–2.26)	5	5.69	0.88(0.29–2.05)
Breast	55	64.59	0.85(0.64–1.11)	1	0.23	4.38(0.11–24.40)	54	64.36	0.84(0.63–1.09)
Genitourinary	106	83.54	1.27(1.04–1.53)	57	33.55	1.70(1.29–2.20)	49	49.99	0.98(0.73–1.30)
Cervix	17	18.18	0.93(0.54–1.50)	–	–		17	18.18	0.93(0.54–1.50)
Uterus	10	9.54	1.05(0.50–1.93)	–	–		10	9.54	1.05(0.50–1.93)
Ovary	6	8.14	0.74(0.27–1.60)	–	–		6	8.14	0.74(0.27–1.60)
Prostate	42	19.38	2.17(1.56–2.93)	42	19.38	2.17(1.56–2.93)	–	–	
Bladder	12	15.29	0.79(0.41–1.37)	9	9.06	0.99(0.45–1.89)	3	6.22	0.48(0.10–1.41)
Kidney	19	13.01	1.46(0.88–2.28)	6	5.11	1.17(0.43–2.56)	13	7.90	1.64(0.88–2.81)
Thyroid	16	11.38	1.41(0.80–2.28)	2	1.50	1.33(0.16–4.81)	14	9.88	1.42(0.77–2.38)
Hematologicmalignancies	26	20.67	1.26(0.82–1.84)	12	9.23	1.30(0.67–2.27)	14	11.44	1.22(0.67–2.05)
All Others	17	18.45	0.92(0.54–1.47)	7	7.83	0.89(0.36–1.84)	10	10.62	0.94(0.45–1.73)

SIR Standardized incidence ratio; CI confidence interval.

### GAD Patients Diagnosed by Psychiatric Specialists

The results for GAD patients diagnosed by psychiatric specialists are shown in Tables 4–5, including SIRs according to age, gender, duration of GAD, and specific cancer types. In this subgroup, there risk for overall cancer or specific cancer types was similar to the general population. Relative to non-psychiatrist-diagnosed GAD patients, this subgroup was younger and had a lower incidence of physical comorbidities, including COPD (males, 22.0 vs. 29.6%; females, 19.0 vs. 22.5%). Thus, the difference in prevalence of comorbidities between psychiatrist- and non-psychiatrist-diagnosed GAD was more pronounced in male patients. Detailed results are shown in Table 6.

**Table 4:** Standardized incidence ratios (SIRs) according to age, gender and duration of psychiatrist-diagnosed generalized anxiety disorder.

	Total			Male			Female		
Characteristics	Observed	Expected	SIR (95% CI)	Observed	Expected	SIR (95% CI)	Observed	Expected	SIR (95% CI)
All cancers	123	118.08	1.04(0.87–1.24)	53	52.70	1.01(0.75–1.32)	70	65.38	1.07(0.83–1.35)
Age, years									
20–39	6	7.38	0.81(0.30–1.77)	1	2.41	0.42(0.01–2.31)	5	4.97	1.01(0.33–2.35)
40–59	43	46.83	0.92(0.66–1.24)	15	17.06	0.88(0.49–1.45)	28	29.76	0.94(0.63–1.36)
60–79	57	53.21	1.07(0.81–1.39)	27	27.09	1.00(0.66–1.45)	30	26.12	1.15(0.77–1.64)
≥80	17	10.66	1.59(0.93–2.55)	10	6.14	1.63(0.78–3.00)	7	4.52	1.55(0.62–3.19)
Duration									
0–1	24	22.29	1.08(0.69–1.60)	14	9.77	1.43(0.78–2.41)	10	12.52	0.80(0.38–1.47)
≥1	99	95.79	1.03(0.84–1.26)	39	42.93	0.91(0.65–1.24)	60	52.86	1.14(0.87–1.46)
1–5	70	66.82	1.05(0.82–1.32)	26	29.80	0.87(0.57–1.28)	44	37.03	1.19(0.86–1.60)
≥5	29	28.97	1.00(0.67–1.44)	13	13.14	0.99(0.53–1.69)	16	15.83	1.01(0.58–1.64)

SIR Standardized incidence ratio; CI confidence interval.

**Table 5:** Standardized incidence ratios (SIRs) for specific cancer types among patients with psychiatrist-diagnosed generalized anxiety disorder.

	Total			Male			Female		
Site of cancers	Observed	Expected	SIR (95% CI)	Observed	Expected	SIR (95% CI)	Observed	Expected	SIR (95% CI)
All cancers	123	118.08	1.04(0.87–1.24)	53	52.70	1.01(0.75–1.32)	70	65.38	1.07(0.83–1.35)
Head and neck	9	10.31	0.87(0.40–1.66)	7	8.27	0.85(0.34–1.74)	2	2.03	0.98(0.12–3.55)
Digestive	35	42.06	0.83(0.58–1.16)	20	23.09	0.87(0.53–1.34)	15	18.97	0.79(0.44–1.30)
Esophagus	1	2.18	0.46(0.01–2.56)	1	1.94	0.51(0.01–2.87)	0	0.23	0.00(0.00–15.94)
Stomach	6	5.49	1.09(0.40–2.38)	5	2.97	1.68(0.55–3.93)	1	2.52	0.40(0.01–2.21)
Colon and rectum	11	16.31	0.67(0.34–1.21)	5	7.58	0.66(0.21–1.54)	6	8.73	0.69(0.25–1.50)
Anus	0	0.12	0.00(0.00–31.47)	0	0.04	0.00(0.00–87.66)	0	0.08	0.00(0.00–49.08)
Liver and biliary tract	15	16.01	0.94(0.52–1.55)	8	9.62	0.83(0.36–1.64)	7	6.39	1.10(0.44–2.26)
Pancreas	2	1.97	1.02(0.12–3.67)	1	0.94	1.07(0.03–5.94)	1	1.03	0.97(0.02–5.41)
Lung andmediastinum	19	13.30	1.43(0.86–2.23)	11	7.30	1.51(0.75–2.70)	8	6.00	1.33(0.58–2.63)
Bone and Softtissue	2	0.98	2.05(0.25–7.40)	2	0.46	4.31(0.52–15.57)	0	0.51	0.00(0.00–7.20)
Skin	1	2.13	0.47(0.01–2.61)	0	0.94	0.00(0.00–3.94)	1	1.20	0.84(0.02–4.66)
Breast	22	16.89	1.30(0.82–1.97)	1	0.06	17.69(0.45–98.54)	21	16.83	1.25(0.77–1.91)
Genitourinary	26	19.72	1.32(0.86–1.93)	10	7.73	1.29(0.62–2.38)	16	11.99	1.33(0.76–2.17)
Cervix	5	4.39	1.14(0.37–2.66)	–	–		5	4.39	1.14(0.37–2.66)
Uterus	4	2.48	1.61(0.44–4.13)	–	–		4	2.48	1.61(0.44–4.13)
Ovary	3	2.14	1.40(0.29–4.10)	–	–		3	2.14	1.40(0.29–4.10)
Prostate	9	4.31	2.09(0.95–3.96)	9	4.31	2.09(0.95–3.96)	–	–	
Bladder	0	3.45	0.00(0.00–1.07)	0	2.16	0.00(0.00–1.71)	0	1.30	0.00(0.00–2.84)
Kidney	5	2.95	1.70(0.55–3.96)	1	1.26	0.79(0.02–4.41)	4	1.68	2.38(0.65–6.09)
Thyroid	2	3.23	0.62(0.07–2.24)	0	0.45	0.00(0.00–8.13)	2	2.78	0.72(0.09–2.60)
Hematologicmalignancies	3	5.02	0.60(0.12–1.75)	2	2.36	0.85(0.10–3.06)	1	2.66	0.38(0.01–2.09)
All Others	4	4.45	0.90(0.25–2.30)	0	2.04	0.00(0.00–1.81)	4	2.40	1.66(0.45–4.26)

SIR Standardized incidence ratio; CI confidence interval.

**Table 6:** Comparison of age and comorbidities between patients with psychiatrist- and non psychiatrist-diagnosed generalized anxiety disorder, stratified by gender.

	Male			Female		
Psychiatrist-diagnosed	Yes	No	*p*-value	Yes	No	*p*-value
No. of patients	2272	4769		3750	9002	
Age at diagnosis(±SD)	45.9(±15.8)	51.8(±16.2)	<0.001	45.7(±15.0)	51.1(±15.3)	<0.001
Comorbidities, n(%)						
Diabetes mellitus	380(16.7)	1015(21.3)	<0.001	622(16.6)	1877(20.9)	<0.001
Dyslipidemia	593(26.1)	1542(32.3)	<0.001	978(26.1)	2803(31.1)	<0.001
Hypertension	685(30.1)	2027(42.5)	<0.001	1012(27.0)	3550(39.4)	<0.001
Ischemic heart disease	498(21.9)	1494(31.3)	<0.001	743(19.8)	2657(29.5)	<0.001
Heart failure	113(5.0)	479(10.0)	<0.001	184(4.9)	843(9.4)	<0.001
Cerebrovascular disease	305(13.4)	925(19.4)	<0.001	464(12.4)	1454(16.2)	<0.001
COPD	500(22.0)	1411(29.6)	<0.001	711(19.0)	2027(22.5)	<0.001
Cirrhosis	67(2.9)	152(3.2)	0.590	65(1.7)	154(1.7)	0.929

SD standard deviation; COPD chronic obstructive pulmonary disease.

## Discussion

To the best of our knowledge, this is the first large population-based study to evaluate the risk of cancer among patients with GAD. This study included 19,793 patients with a follow-up of 89,485 person-years. The data showed an increased overall cancer risk in GAD patients (SIR 1.14) relative to the general population, especially males and patients aged 80 years and older. Specifically, the risks of lung and prostate cancer were increased in male patients. It is interesting to note that cancer risk was not elevated in psychiatrist-diagnosed GAD patients.

The current study design included unbiased subject selection and SIR estimations with matching for age, gender, and calendar year. Because participation in the NHI is mandatory and all residents of Taiwan can access healthcare with low co-payments, referral bias is essentially removed and follow-up is complete. To apply for a cancer catastrophic illness certificate, pathologic proof of malignancy is mandatory, and laboratory and imaging studies should also be provided. Patients with a certificate of catastrophic illness can be exempted from related medical expenses, especially hospital costs. Therefore, cancer diagnoses in this study were not only reliable, but also exhaustive.

While some large retrospective studies have investigated the association between anxiety and cancer risk, their results were inconsistent. [20]–[22] A study of 24,066 patients in Taiwan with a follow-up of 193,207 person-years reported an increased risk of developing prostate cancer and a marginally decreased risk of cervical cancer, but no increase in overall cancer risk. [20] In addition, a study of 24,290 patients in the UK showed higher risks for lung and brain cancer, but no increase in overall cancer risk. [21] A prospective study of 10,892 women in Finland with a follow-up of 6–9 years showed that anxiety was not a risk factor for breast cancer. [22] These divergent results may be due to different definitions of anxiety and inclusion criteria. The Taiwan study included patients with GAD, panic disorder, agoraphobia, social phobia, post-traumatic stress disorder (PTSD) and obsessive-compulsive disorder (OCD), selecting only subjects who had been admitted at least three times. These inclusion criteria may have predominantly selected patients with severe illness and certain types of anxiety disorders, such as PTSD and OCD, as most GAD patients are treated in outpatient settings. [12] Similarly, the UK study might have also assessed severely ill patients, as it included patients who were admitted with ICD-9 code 300 (anxiety, dissociative and somatoform disorders), ICD-8 code 300 (neurosis), ICD-7 code 310 (anxiety reaction without mention of somatic symptoms), or ICD-10 code F41 (other anxiety disorders, including panic disorder without agoraphobia, generalized anxiety disorder, other mixed anxiety disorders, other specified anxiety disorders, and anxiety disorder, unspecified). In addition, the use of different ICD systems and several ICD codes made the study group heterogeneous. In the Finland study, anxiety was evaluated by the Spielberger State-Trait Anxiety Inventory, rather than physician diagnosis. Thus, the inconsistency of results across these studies could be due to different definitions of anxiety and inclusion criteria. The current study had a more homogenous population, consisting of only GAD patients.

The current study found that overall cancer risk in GAD patients was higher than the general population with an SIR of 1.14. It has been hypothesized that psychological factors including anxiety could impair immune and endocrine function. [27], [28] Anxiety activates the hypothalamic-pituitary-adrenal (HPA) axis and the sympathetic-adrenal-medullary axis, elevating corticosterone and other stress hormones, such as catecholamines. These hormones could modulate the activity of the tumor microenvironment. [29] Many studies have shown that stress can affect the cellular process involved in the repair of damaged DNA, [30] which could facilitate development of oncogenic mutations. Stress hormones could also accelerate growth of tumor cells by altering glucose uptake, [31] promote tumor migration by increasing the expression of matrix metalloproteinase-2, [32] and stimulate angiogenesis by inducing production of pro-angiogenic cytokines, such as vascular endothelial growth factor and interleukin-6. [29] These mechanisms might contribute differently to specific stages of cancer development and to certain cancer types. However, the median follow-up in the current study was 4.34 years, which may have been too short to detect increased risk of some malignancies, especially for cancers with long latency periods.

In the present study, incident cancers were increased in the first year after diagnosis of GAD with an SIR of 1.22. One possible explanation for this result is surveillance bias. [33] Patients with GAD are likely to have more outpatient visits and thus more medical examinations than the general population, which leads to an earlier diagnosis of cancer. In addition, the close temporal relationship between diagnosis of GAD and cancer could support anxiety as a paraneoplastic manifestation in patients with undiagnosed malignancy. [34], [35] While excluding data from patients for whom GAD and cancer were diagnosed within one year decreased the SIR to 1.12, it remained significantly elevated relative to the general population. Hence, it seems reasonable to conclude that increased cancer risk in GAD patients in the current study was not due only to surveillance bias or a paraneoplastic manifestation.

The current study also found an increased SIR of 1.56 in GAD patients aged 80 and older, especially in males. Given that the risk of prostate cancer was also increased significantly, it is reasonable to infer that such increased SIR was due mainly to prostate cancer, which is commonly diagnosed in advanced age. [36], [37] Because prostate cancer usually has an indolent course and can be detected with by rectal examination and a prostate specific antigen (PSA) test, it is prone to surveillance bias. The Taiwan study showed that a higher proportion of patients with anxiety disorder underwent a PSA test, and they had a higher risk of prostate cancer. [20] Therefore, the increased risk of prostate cancer in patients with anxiety disorders may be mainly due to surveillance bias. However, SIRs of prostate cancer were still increased when the person-time of the first year was excluded, suggesting a relationship between GAD and prostate cancer. One possible explanation for the increased risk of prostate cancer in GAD patients is that obesity, lack of exercise, and high blood pressure are more frequent in patients with anxiety disorders than in the general population, [8], [38], [39] and these characteristics increase the risk of prostate cancer. [40]–[42].

The current study found an increased risk of lung cancer in male patients. Smoking is a possible confounder, as GAD is associated with heavy smoking, nicotine dependence, and smoking cessation failure, and smoking itself is a major risk factor for lung cancer. [43]–[45] However, the risks of other smoking-related malignancies, such as head and neck cancer, esophageal cancer and bladder cancer, were not increased in the study cohort. Another possible explanation for the increased risk of lung cancer in GAD patients could be that patients with lung diseases, such as COPD, are prone to anxiety, [11] and these lung diseases themselves are risk factors for lung cancer. [46] Furthermore, these lung diseases have a higher incidence in males, [47]–[49] which could explain why increased lung cancer risk was noted in male GAD patients, but not females.

While the overall GAD cohort had an increased risk of cancer, prostate cancer, and lung cancer, no change in risk was observed in the subgroup of psychiatrist-diagnosed GAD patients. This inconsistency might be due to a smaller number of patients in the psychiatrist-diagnosed cohort, which decreased statistical power for this group. Furthermore, as shown in Table 6, non-psychiatrist-diagnosed GAD patients had more physical comorbidities, such as COPD. Many physical comorbidities have been demonstrated to increase cancer risk. [46], [50], [51] For example, COPD is a risk factor for lung cancer. Therefore, the increased risk of lung cancer in GAD patients could be due to physical comorbidities, such as COPD.

There are several limitations to this large population-based study. First, many demographic variables were not available, such as family history of cancer, environmental exposures, diet, cigarette smoking, and alcohol use. Second, the diagnostic accuracy and severity of GAD could not be obtained. Whether the severity of GAD is a related to cancer development should be further evaluated. Third, this study did not assess the effect of psychotropics on cancer risk. Antipsychotics and benzodiazepines are two main treatments for GAD. Antipsychotics have been hypothesized to account for reduced cancer risk in schizophrenic patients, and benzodiazepines have been proposed to increase cancer risk. [52], [53] Finally, the follow-up duration of the current study might be too short to detect carcinogenesis of certain types of cancers.

In conclusion, this population-based retrospective cohort study found increased cancer risk among patients with GAD, especially males, compared with the general population. Specifically, the risks of prostate and lung cancers among male GAD patients were significantly increased. However, cancer risk did not increase in psychiatrist-diagnosed GAD patients. Increased cancer risk in GAD patients may be due to physical comorbidities and surveillance bias. Further large, unbiased population-based prospective studies are needed to investigate the association between GAD and cancer risk.
